# Diagnosis and treatment of traumatic duodenal rupture in children

**DOI:** 10.1186/s12876-022-02136-w

**Published:** 2022-02-12

**Authors:** Yuanyuan Luo, Xiaobing He, Lanlan Geng, Runxian Ouyang, Yingyi Xu, Yufeng Liang, Jinhui Wu, Hong Zhang, Zhihua Ye, Rongjun Zou, Qiang Wu, Chengwei Chai

**Affiliations:** 1 Department of Gastrointestinal Surgery, Guangzhou Women and Children’s Medical Center, Guangzhou Medical University, No. 9 Jinsui Road, Zhujiang New Town, Tianhe District, Guangzhou City, 510623 Guangdong Province China; 2 Guangdong Provincial Key Laboratory of Research in Structural Birth Defect Disease, Guangzhou Women and Children’s Medical Center, Guangzhou Medical University, Guangzhou City, 510623 China; 3 Department of Anaesthesiology, Guangzhou Women and Children’s Medical Center, Guangzhou Medical University, Guangzhou City, 510623 China; 4 Department of Paediatric Intensive Care Unit, Guangzhou Women and Children’s Medical Center, Guangzhou Medical University, Guangzhou City, 510623 China; 5 Department of Paediatric Nutrition, Guangzhou Women and Children’s Medical Center, Guangzhou Medical University, Guangzhou City, 510623 China; 6 Heart Center, Guangdong Provincial Key Laboratory of Research in Structural Birth Defect Disease, Guangzhou Women and Children’s Medical Center, Guangzhou Medical University, Guangzhou City, 510623 China

**Keywords:** Duodenal rupture, Children, Multidisciplinary, Diagnosis, Surgery, Postoperative management

## Abstract

**Background:**

The purpose of this study was to investigate the diagnosis and treatment experience of traumatic duodenal ruptures in children.

**Methods:**

Clinical data were collected from four children suffering from a traumatic duodenal rupture who were admitted to and treated by our hospital from January 2012 to December 2020. The early diagnosis and treatment, surgical plan, postoperative management, complications, and prognosis of each child were analyzed. The key points and difficulties of the diagnosis and treatment for this type of injury are summarized.

**Results:**

One child had an extreme infection caused by drug-resistant bacteria, which resulted in severe complications, including wound infection, dehiscence, and an intestinal fistula. One child developed an anastomotic stenosis after the duodenostomy, which improved following an endoscopic balloon dilatation. The other two children had no relevant complications after their operations. All four patients were cured and discharged from hospital. The average hospital stay was 48.25 ± 26.89 days. The follow-up period was 0.5 to 1 year. No other complications occurred, and all children had a positive prognosis.

**Conclusions:**

The early identification of a duodenal rupture is essential, and surgical exploration should be carried out proactively. The principles of damage-control surgery should be followed as much as possible during the operation. Multidisciplinary cooperation and management are both important to reduce the occurrence of postoperative complications and improve cure rates.

## Background

Trauma is the leading cause of death in children under 14 years of age. Blunt abdominal trauma usually causes closed injuries in children due to their thin abdominal wall, weak muscle development, and vulnerable abdominal organs. These injures commonly affect solid organs including liver, spleen, kidney, pancreas, etc., and hollow organs including the small intestine, colon, stomach, etc. Due to the unique anatomical location of the duodenum, a traumatic rupture is rare, and accounts for only 2–10% of closed abdominal injuries in children [1]. However, as one of the unique physiological characteristics of the duodenum, it contains irritating digestive fluid. Once it ruptures, the mortality rate can be as high as 19%. Delayed diagnosis and treatment or inappropriate treatment can increase the mortality rate [2]. Therefore, the effective diagnosis and treatment of traumatic duodenal ruptures in children is not only a challenge to surgeons but also requires collaboration from multiple disciplines, including emergency, imaging, anesthesiology, gastroenterology, nutrition, rehabilitation, and psychology departments along with the nursing team. This study aimed to analyze the clinical data of four children with traumatic duodenal ruptures who were admitted to and treated in our hospital between January 2012 and December 2020. Along with a thorough comparison to the literature, the current study analyzed and summarized the experience of the multidisciplinary diagnosis and treatment of traumatic duodenal ruptures in children. This report was completed with the informed consent from family members of all patients and the approval of the hospital ethics committee.

## Methods

### General patient data

**Fig. 1:**
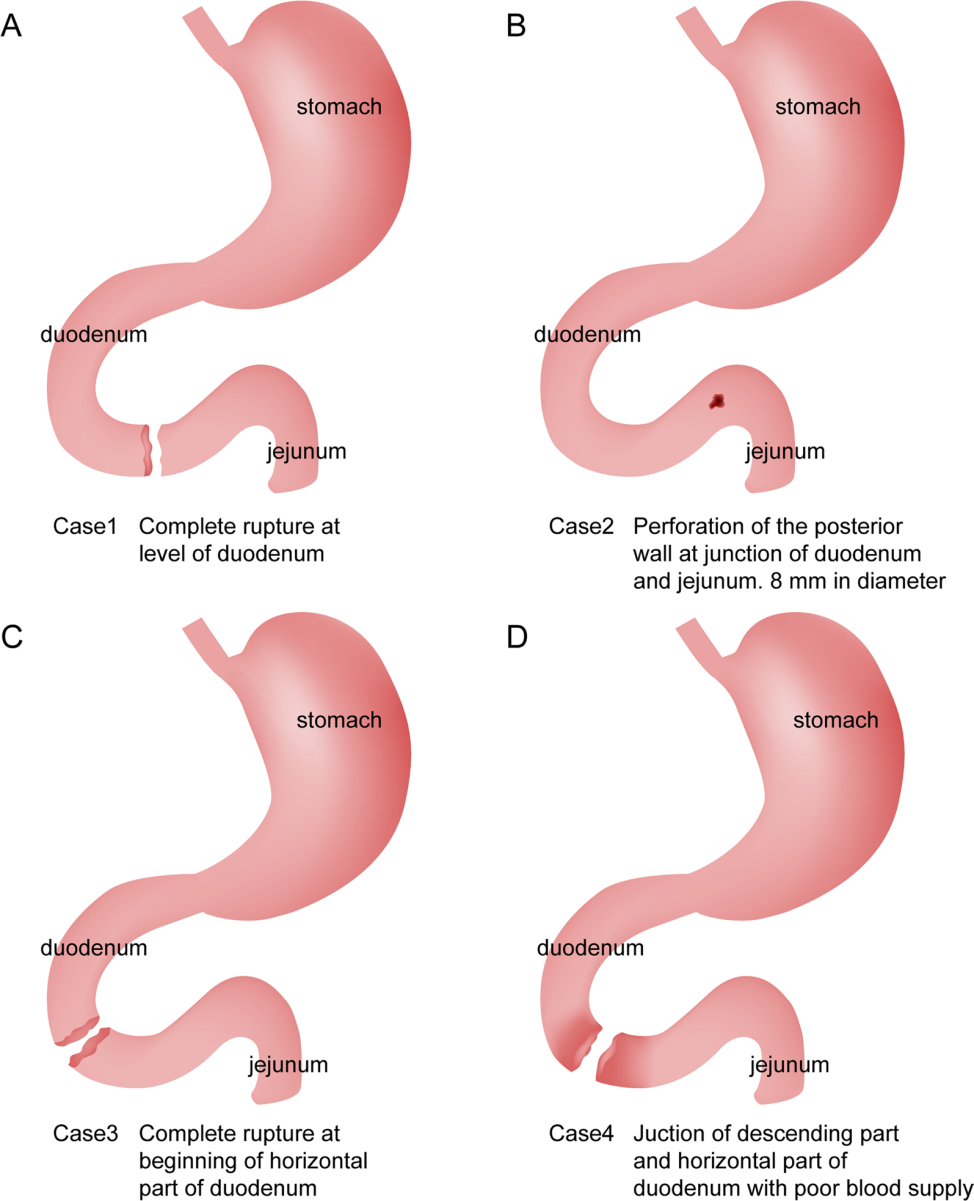
The site of the rupture or perforation of the duodenum in each case.** A** Case 1, Complete rupture at level of duodenum.** B** Case 2, Perforation of the posterior wall at junction of duodenum and jejunum. 8 mm in diameter.** C** Case 3, Complete rupture at beginning of horizontal part of duodenum.** D** Case 4, Junction of descending part and horizontal part of duodenum with poor blood supply

From January 2012 to December 2020, a total of four children with traumatic duodenal ruptures were admitted to and treated at our hospital. There were three boys and one girl with an average age of 4.1 years (range 1–9 years). All patients were transferred from other hospitals. One patient was crushed by a heavy object and was transferred to our hospital 1 day after the initial injury (Case 1). One patient was physically injured by another person (punched, kicked) and was transferred to our hospital 3 days after the initial injury (Case 2). One patient was injured in a motor vehicle collision and had undergone surgical exploration at another hospital but was transferred to our hospital 2 days after the injury (Case 3). One patient fell from a bicycle and was crushed by a handlebar of the bicycle. This patient had undergone multiple surgeries and conservative treatments and was transferred to our hospital 42 days after the initial injury due to his deteriorated condition (Case 4). All children in this study underwent surgical treatment at our hospital. In two patients, the perforation sites were located at the horizontal aspect of the duodenum, in one patient at the junction of the descending aspect and the horizontal section of the duodenum, and the final patient at the junction of the duodenum and jejunum (Table 1; Fig. 1).

**Table 1:** Clinical data of four children with traumatic duodenal rupture

Case	Age (years)	Sex	Cause of injury	Clinical manifestations	Abdominal tenderness	Laboratory examination	Radiography (free air under the diaphragm)	B-mode ultrasound (effusion)	CT	Treatments in another hospital	Time to operation in our hospital after injury (days)
1	1.5	Male	Oxygen cylinder crush	Abdominal pain	+	+	-	+	+	No	2
2	3	Male	Physically injured by another person	Abdominal pain, nausea, vomiting, sluggishness	+	+	+	+	+	Anti-shock, ventilator-assisted ventilation	3
3	3	Female	Motor vehicle accident	Abdominal pain	+	+	-	+	+	Exploratory laparotomy on the first day after injury	2
4	9	Male	Fall from bike	Abdominal pain, nausea, vomiting	+	+	-	+	+	Exploratory laparotomy + right ureteral stent implantation on the day of injury; exploratory laparotomy + duodenal repair + three-tube drainage on the next day; intestinal fistula and ureteral fistula occurred after surgery; conservative treatment for 42 days	46

Case	Age (years)	Sex	Rupture site	Concomitant injury	Grades of duodenal injury	Surgical approach	Postoperative complications and treatment	Hospital stay (days)	Results
1	1.5	Male	Complete rupture at level of duodenum	No	III	Duodenostomy + gastrostomy + jejunostomy	No	28	Cured
2	3	Male	Perforation of the posterior wall at junction of duodenum and jejunum, 8 mm in diameter	No	II	Repair of perforation of posterior duodenal wall	No	30	Cured
3	3	Female	Complete rupture at beginning of horizontal section of duodenum	No	III	Duodenostomy + jejunostomy	Fungal infection, drug combination; anastomotic stenosis, endoscopic anastomotic dilation	49	Cured
4	9	Male	Junction of descending section and horizontal section of duodenum completely ruptured with poor blood supply	Rupture of the right ureter	V	Duodenostomy + gastrostomy + jejunostomy	Multidrug-resistant bacterial and fungal infections, symptomatic and combination medication; poor wound healing, wound care; intestinal fistula, conservative treatment	86	Cured

### Diagnosis and treatment

All children in this study had a history of severe abdominal pain following injury, and two experienced accompanying nausea and vomiting. All had signs of abdominal tenderness. The lab results showed elevated levels of C-reactive protein, white blood cells, neutrophils, α-amylase, lipase, and lactate dehydrogenase. Abdominal radiography showed free air under the diaphragm in one patient. An abdominal B-mode ultrasound revealed abdominal effusion. Abdominal computed tomography (CT) showed retroperitoneal air. Prior to the operation, two patients had undergone surgical treatments at other hospitals, where the diagnosis of duodenal rupture had been confirmed. The duodenal rupture was suspected in the other two patients before the operation and was confirmed during surgical exploration. All four children underwent one-stage duodenal repair. Concurrent jejunostomy and gastrostomy were performed in three and two patients, respectively (Table 1).

## Results

According to the classification of duodenal injury (Table 2) established by the American Association for the Surgery of Trauma [3], the traumatic duodenal rupture was classified as a grade II injury in one patient (25%), grade III injury in two patients (50%), and grade V injury in one patient (25%). One child was transferred to our hospital due to deteriorated conditions after two operations and 42 days of conservative treatment at another hospital. This patient underwent emergency surgery due to active bleeding and had postoperative complications, including infection caused by drug-resistant bacteria, wound infection, dehiscence, and an intestinal fistula. One child developed an anastomotic stenosis following the duodenostomy, which was improved by endoscopic balloon dilatation. No related complications occurred in the other two children. All four patients were cured and discharged from the hospital. The average hospital stay was 48.25 ± 26.89 days. The follow-up period was 0.5–1 year. No other complications occurred, and their prognoses were good (Table 1).

**Table 2:** Classification of duodenal injury [3]

Grades	Injury description
I	Hematoma: involving one part of the duodenum
	Laceration: partial thickening of the intestinal wall without perforation
II	Hematoma: involving more than one part of the duodenum
	Laceration: < 50% of the circumference
III	Laceration: 50–70% of the circumference of D2 and/or
	50–100% of the circumference of D1, D3, D4
IV	Laceration: > 75% circumference of D2
	Involvement of the ampulla or distal common bile duct
V	Laceration: complete rupture of the pancreaticoduodenum
	Blood vessels: decreased blood supply to the duodenum

## Discussion

The anatomy of the duodenum is unique. The first segment (bulb) is an intraperitoneal organ with a certain degree of mobility, while the other segments are retroperitoneal organs with relatively fixed positions. Blunt violence can directly press the duodenum against the spine and injure it or cause a sudden closure of the pylorus and the duodenojejunal flexure to form a closed intestinal segment, and the sharply increased pressure within the segment can cause the duodenum rupture [4]. Due to the deep anatomical position of the duodenum, typical symptoms and signs of acute peritonitis are often lacking after trauma, and it is easy to miss the diagnosis or misdiagnose the injury in clinical practice. Duodenal ruptures are associated with high rates of complications and mortality. The effective diagnosis and treatment of duodenal ruptures cannot be completed by a single discipline, but rather it is a sequential and comprehensive process, including early identification and diagnosis, selection of treatment/surgical options, and postoperative management involving the collaboration of a multidisciplinary team.

### Early diagnosis of duodenal rupture

Early diagnosis is a key step in the successful treatment of duodenal ruptures in children, and it affects the survival rate even more than the nature of the injury itself. Delayed diagnosis is a direct factor leading to an increased mortality rate [2, 5]. Of the four children in this study, two were diagnosed and treated early, and the other two had delays in diagnosis and treatment. The latter two patients had a significantly longer hospital stay than the patients with early diagnoses. Moreover, they had various degrees of complications during the recovery period, which included severe infection caused by drug-resistant bacteria, wound infection, dehiscence, and an intestinal fistula in one patient, and an anastomotic stenosis after duodenostomy in the other patient.

Compared with rupture of the duodenum in adults, duodenal ruptures in children are primarily caused by closed injuries rather than penetrating injuries. The fact that most children cannot accurately describe the injury history or symptoms makes an early diagnosis more difficult. During the first encounter with a child suffering from blunt abdominal injury, the physician in the outpatient or emergency service should not underestimate the likelihood or severity of a duodenal rupture. Careful history collection and physical examination are necessary. In this population, approximately 90% of children present with some nonspecific positive signs, such as abdominal pain and vomiting, and approximately one third of children present with signs of peritonitis at an early stage. These indicate emergency surgical exploration to achieve timely intraoperative diagnosis and treatment [6]. Some children with pancreatic injuries may present with significant increases in blood and urine amylase and lipase, but the laboratory results are not specific and cannot be used to determine whether there is any injury to the intestine [7]. Abdominal CT scans have higher sensitivity and specificity for the early diagnosis of duodenal injuries [8] and can increase the diagnosis rate to 84.21% [9]. Importantly, CTs can show free air and effusion in the right retroperitoneal space and the hepatorenal recess in the patients with severe injury or comorbid pancreatic injury, in addition to directly showing the interrupted continuity of the injured duodenum, thickening of the intestinal wall, or air accumulation around the duodenum [10].

However, it is unreasonable and unrealistic to use abdominal CTs as a first-line diagnostic tool for all children with closed abdominal injuries. In contrast, abdominal ultrasound is a simpler and easier procedure for initial screenings. For children with a closed abdominal trauma and stable hemodynamics, Karam et al. have proposed a scale, the Blunt Abdominal Trauma in Children (BATiC; Table 3), to determine the necessity of emergency CT or temporary observation [11]. The scale consists of 10 simple and easy-to-collect variables, including abdominal signs, laboratory test results, abdominal ultrasound results, and others, which are assigned different scores. The sum of these scores is the BATiC score.

**Table 3:** Blunt Abdominal Trauma in Children (BATiC) scale [11]

Variable	Reference value	Score
Ultrasound	+	4
Abdominal pain	+	2
Signs of peritoneal irritation	+	2
Hemodynamic disorders	+	2
AST	> 60 U/L	2
ALT	> 25 U/L	2
WBC	> 9.5 10^9^/L	1
LDH	> 330 U/L	1
LIPA	> 30 U/L	1
Cr	> 50 μg/L	1

AST, aspartate aminotransferase; ALT, alanine aminotransferase; WBC, white blood cell; LDH, lactate dehydrogenase; LIPA, lipase; Cr, creatinine

A BATiC score of seven points or less indicates a small likelihood of abdominal organ injury, and clinical observation is recommended. However, in the early stage of injury, some variables may not represent the severity of the injury well, so it is necessary to admit children for further dynamic observation. Repeated examinations and multiple evaluations of the BATiC score is necessary to prevent a missed diagnosis of a duodenal rupture [12].

For critically ill children with closed abdominal injuries who had respiratory and circulatory disorders at the early stage of injury, outpatient and emergency physicians should not ignore the possibility of a duodenal rupture. Progressive anti-shock treatment should be performed to maintain vital signs. Meanwhile, a quick and accurate diagnosis of the injury is necessary. Once the duodenal rupture is diagnosed or cannot be ruled out, surgical exploration should be proactively performed.

### Surgical exploration and treatment of duodenal ruptures

For children who are highly suspected of having a duodenal rupture, an exploratory laparotomy should be performed early to avoid the serious consequences of a delayed diagnosis. Most of the children have a concurrent organ injury. If a surgeon ignores the retroperitoneal exploration of the duodenum due to the findings of an organ injury that are easily found in the exploration, this may result in a missed diagnosis. In the first exploration, the missed diagnosis rate of duodenal ruptures can be as high as 18.75% [13]. For example, the most critically ill child in this study (Case 4) underwent an initial surgical exploration at another hospital, which only found a ureteral injury. The urethral injury was managed accordingly, but the child’s condition rapidly worsened the next day. A second operation had to be performed, which then revealed the duodenal rupture. Therefore, if intraoperative exploration finds bile staining in the posterior peritoneum or edema, effusion, congestion, fat necrosis, or crepitus in the posterior peritoneum and the right mesocolon, duodenal rupture should be highly suspected [14]. A Kocker incision should be made with division of the Trietz ligament (if necessary) to fully expose each segment of the duodenum for further examination. The surgeon may need to ask a circulating nurse to inject color reagent through the gastric tube and then observe whether any leakage of the color reagent is present around the duodenum. Thorough and careful exploration is critical.

The main surgical treatment method is selected based on the comprehensive assessment of the location, severity, and combined injury of the duodenal rupture, as well as the general condition of the patient. Simple repair of the duodenal rupture is suitable for patients within 10 h post-injury or when the rupture is small with smooth edges and local, mild edema. Roux-en-Y anastomosis between the duodenum and the jejunum is suitable for children with large rupture defects, mild local edema, and excessive tension if performing a simple repair. Duodenal diverticulization, namely repair of the rupture, removal of the distal stomach, and a Billroth II gastrectomy, is suitable for critically ill children who have severe contamination at the rupture site and in the abdominal cavity. Pancreaticoduodenectomy is suitable for children with severe pancreatic and biliary injuries [15]. However, for children with severe injuries and poor general condition, one-stage surgical treatment should not be pursued due to the complicated, time-consuming, and invasive surgical procedures that will aggravate the condition or lead to intolerance to surgery [16] and a higher incidence (up to 67%) of serious postoperative complications [17]. Some complications may become life-threatening.

In recent years, some scholars have put forward the concept of damage-control surgery (DCS) for patients with severe duodenal injury (grade V), which has changed treatment strategies for severely injured children. The concept of DCS focuses on reducing any further damage caused by the operation itself and advocates simple approaches to prevent aggregation of trauma and create conditions for recovery and treatment after severe trauma. The treatment principle includes three steps: rapid control of the injury (rapid closure of the rupture and full drainage); resuscitation (stabilization of the internal environment and systemic hemodynamic indicators); and definitive surgery (digestive tract reconstruction) [18]. For severely ill children with indications for DCS, it is recommended to follow the DCS principle. The operation should be as simple and reasonable as possible, with the main purpose of saving the life and preventing any aggravation of the trauma, especially in hospitals with limited medical resources. For example, the most critically ill child (Case 4) in this study was transferred to our hospital 42 days after the initial injury and had already undergone two major surgeries. He was suffering from severe infection, disturbance of homeostasis, coagulation dysfunction, and severe malnutrition. The child had an extremely poor general condition and could not tolerate another operation immediately. Therefore, initial treatments focused on the symptoms, and included control of the infection, adequate abdominal drainage, and nutritional support, to foster the conditions for reoperation. Although the child had an emergency operation due to active bleeding on the fourth day after admission, it was undeniable that the child’s internal environment and nutritional status had greatly improved after 4 days of active symptomatic treatment. This provided the foundation for the child to tolerate the reoperation and achieve a stable recovery after the reoperation. Another child (Case 3) in this study had undergone surgical exploration at another hospital, which suggested the possibility of a duodenal rupture. Due to their lack of experience in pediatrics, they terminated the exploration without further treatment and immediately transferred the child to our hospital. After admission, the patient’s condition was fully evaluated, a radical operation was performed immediately, and the child’s postoperative recovery was satisfactory. In this respect, when considering whether to choose a DCS treatment plan, in addition to considering the severity of the injury, the ability of the hospital to manage the condition and desire to solve the problem immediately should also be considered. The concept of DCS has not been strongly promoted, but the clinical application of this theory is a leap forward in the development of trauma surgery. Its role will continue to evolve with further research on the exact resuscitation indications, timing, and surgical techniques [19].

Regardless of the surgical plan chosen, adequate drainage after surgery is necessary. Effective decompression in the stomach and duodenum is an important factor in preventing duodenal fistulas and ensuring the recovery of the patient. The indwelling of the jejunostomy tube also plays an important role in postoperative nutritional recovery. An indwelling drainage tube in the abdominal cavity is also conducive to the observation of anastomotic healing and abdominal infection after the operation.

### Postoperative management of duodenal ruptures

Children with traumatic duodenal ruptures have a high risk of septic shock due to severe trauma and severe abdominal cavity contamination. There is currently a lack of literature regarding children-centered treatment plans or surgical methods. Neither a recognized diagnostic nor treatment process for reference is available. Therefore, early and empirical treatment with broad-spectrum antibiotics is necessary, for instance, with imipenem and cilastatin sodium. In some children, dual and triple antibiotics, or antibiotics for resistant bacterial infections may be required based on bacterial cultures and drug sensitivity tests. During treatment, the clinical pharmacy team should be consulted to monitor and adjust the antibiotic treatment plan according to the patient’s condition. In the middle and late stages of treatment, two patients in this study developed fungal infections, so the antifungal drug voriconazole was added to the treatment regimen. The fungal infections may have been related to the children’s severe illnesses, low immunity, and long-term use of antibiotics, which can result in infections caused by conditional pathogens. Therefore, for these severely ill children, fungal infections are also a potential condition that cannot be ignored.

The unique anatomical and physiological characteristics of the duodenum are that it contains a large amount of irritating digestive fluid, so adequate drainage is an important approach to treat duodenal ruptures. Therefore, multiple drainage tubes, including a gastrostomy tube, duodenal drainage tube, jejunal nutrition tube, abdominal drainage tube, and pelvic drainage tube, are placed in such children after surgery. Taking care of these tubes requires a highly diligent and skilled nursing team. In addition to daily care of the tubes and prevention of accidental fall-out of the tubes, it is necessary to observe and record all properties and quantities of the drainage fluid, and changes to each tube in detail since the loss of digestive fluid has a great impact on the internal environment of the body.

To promote anastomotic healing, the children are required to fast for more than 1 week after the surgery. Meanwhile, somatostatin and proton pump inhibitors should be used to inhibit the secretion of various digestive fluids. During the fasting period, total parenteral nutrition is started and then gradually shifted to enteral nutrition according to the patient’s condition. This process requires the participation of the nutritionist team to adjust and formulate individualized nutrition plans at the correct times according to the caloric needs of the patients at different stages of recovery. For example, the most critically ill child in this study (Case 4) had undergone many major operations and had an extremely poor nutritional status during his transition to enteral nutrition. Therefore, the commonly used amino acid-based formula in the transitional phase could no longer meet his caloric needs for recovery. The nutritionist team proposed an individualized feeding plan that incorporated donated breast milk. The proposal of this individualized plan and the adjustment of the subsequent nutritional plan played an important role in the postoperative recovery of the child. The weight of the child increased from 15 kg at the time of admission to 27 kg at the time of discharge.

**Fig. 2:**
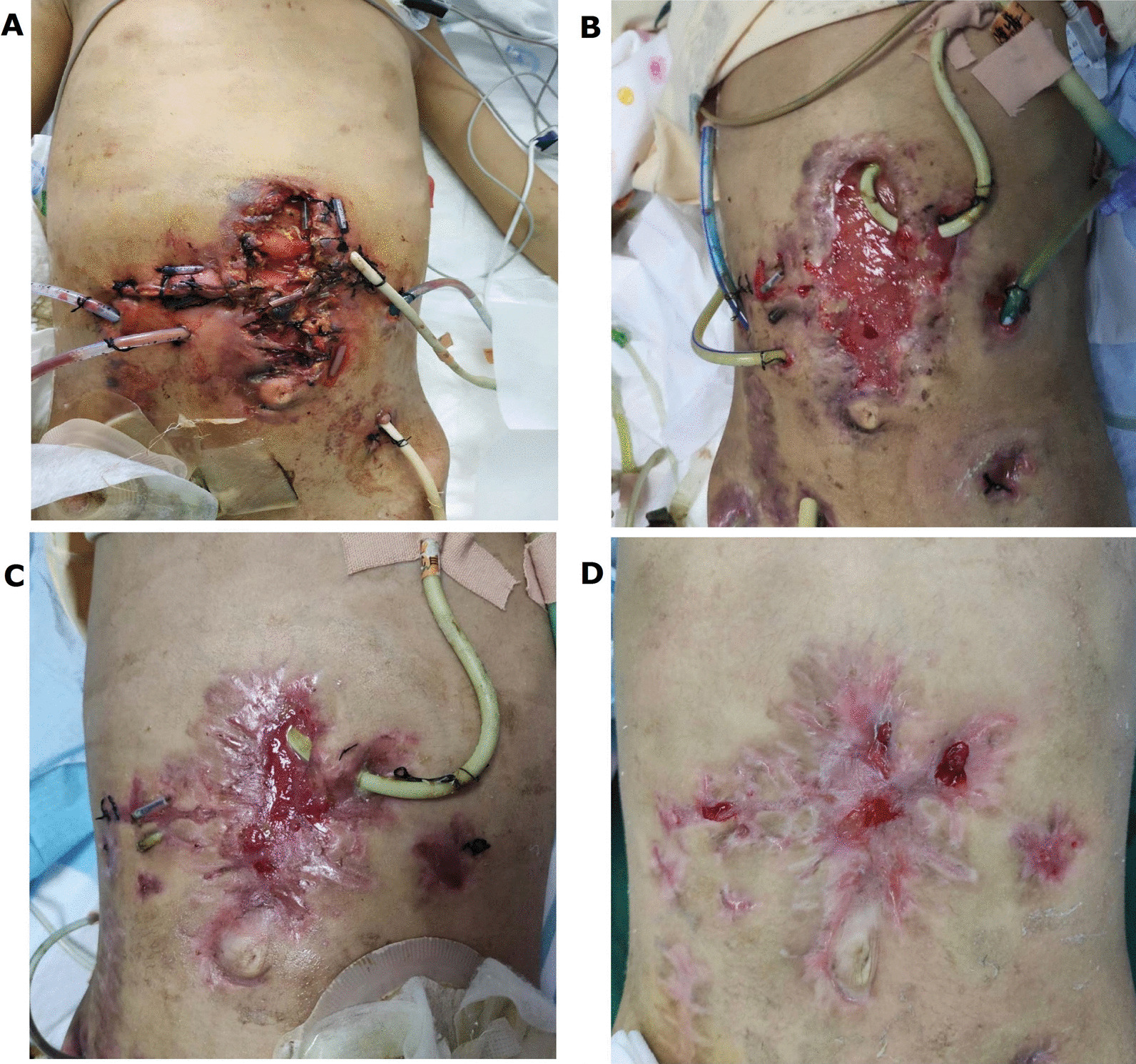
The pictures show the abdominal wound healing in of Case 4 case. **A** On the 5th day after the operation, the abdominal incision began to ulcerate and split widelyopen. **B** On the 20th day after surgery, the incision did not deteriorate further, and a large amount of fresh granulation tissue growth was seen under the treatments, including negative pressure drainage and special dressings. **C** On the 45th day after the operation, the incision continued to heal, and fresh granulation tissue can be seen in the central part section and with some surrounding scar tissue around it were visible. **D** On the 58th day after the operation, the incision was basically had healed considerably

Children with traumatic duodenal ruptures often have serious contamination of the abdominal cavity and require long-term indwelling of multiple drainage tubes after surgery. Particularly, children who have undergone multiple operations are prone to poor wound healing. During the operation, surgeons should use tension-relieving sutures to prevent the occurrence of this complication as much as possible. Post-operation, the care team should closely monitor the wound healing in the early stage and quickly manage any problems observed. For example, the most critically ill child in this study (Case 4) had had extensive ulceration and dehiscence that began the fifth day after surgery. The wound care team found the problem quickly and adopted various methods such as negative-pressure drainage and special dressings to effectively prevent further deterioration of the wound condition and avoid the risk of reoperation. The wound healing had reached grade A when the child was discharged from the hospital (Fig. 2).

Anastomotic healing is a postoperative focus. In this study, the anastomoses healed well without complications in three patients. However, one child (Case 3) had an anastomotic stenosis after the operation and could not eat large, difficult-to-digest foods in the early stages of recovery. The gastroenterology team performed an endoscopic balloon dilatation for the child 2 weeks and 1.5 months (post-discharge) after the operation with satisfactory results. The child could eat and normally drink after treatment, and the repeated gastrointestinal angiography did not show any sign of anastomotic stenosis. In addition, due to the serious condition and long recovery in these children, they presented with varying degrees of psychological disorders and muscle atrophy from disuse during recovery. Thus, teams from the Department of Psychology and the Department of Rehabilitation were also part of the treatment group.

Importantly, the small sample size lowered the power of the present study. Therefore, additional new cases are still being collected to accumulate more evidence to support diagnosis and treatment processes for this condition. Still, controlling any infection, adequate drainage, and nutritional support are three primary tasks in the postoperative management of children with duodenal ruptures, but details that are easily overlooked during the treatment process are also a focus. Especially for the treatment of severely ill patients, multidisciplinary teams are necessary.

## Conclusions

Traumatic duodenal ruptures in children are rare in clinical practice but can cause critical illnesses and are associated with high mortality rates once they occur. Doctors need to race against time for early identification, diagnosis, and treatment. In the case of a highly suspected duodenal rupture, an exploratory laparotomy should be performed early and comprehensively to avoid a missed diagnosis. For critically ill children, it is recommended to follow the DCS principle, and the operation should be as simple and reasonable as possible, with the main purpose of saving the life and preventing any aggravation of trauma. Postoperative management includes the three main tasks of eliminating any risk of infection, adequate drainage, and nutritional support, as well as other management details that are easily overlooked. The multidisciplinary model should be practiced throughout the treatment process.

## Data Availability

The datasets used and/or analyzed during the current study are available from the corresponding author on reasonable request.

## Abbreviations

CT: Computed tomography
BATiC: Blunt Abdominal Trauma in Children
DCS: Damage-control surgery
